# Comprehensive Risk System Based on Shear Wave Elastography and BI-RADS Categories in Assessing Axillary Lymph Node Metastasis of Invasive Breast Cancer—A Multicenter Study

**DOI:** 10.3389/fonc.2022.830910

**Published:** 2022-03-10

**Authors:** Huiting Zhang, Yijie Dong, Xiaohong Jia, Jingwen Zhang, Zhiyao Li, Zhirui Chuan, Yanjun Xu, Bin Hu, Yunxia Huang, Cai Chang, Jinfeng Xu, Fajin Dong, Xiaona Xia, Chengrong Wu, Wenjia Hu, Gang Wu, Qiaoying Li, Qin Chen, Wanyue Deng, Qiongchao Jiang, Yonglin Mou, Huannan Yan, Xiaojing Xu, Hongju Yan, Ping Zhou, Yang Shao, Ligang Cui, Ping He, Linxue Qian, Jinping Liu, Liying Shi, Yanan Zhao, Yongyuan Xu, Yanyan Song, Weiwei Zhan, Jianqiao Zhou

**Affiliations:** ^1^ Department of Ultrasound, Ruijin Hospital, Shanghai Jiaotong University School of Medicine, Shanghai, China; ^2^ Department of Medical Ultrasound, Yunnan Cancer Hospital & The Third Affiliated Hospital of Kunming Medical University, Kunming, China; ^3^ Department of Ultrasound in Medicine, Shanghai Jiao Tong University Affiliated Sixth People’s Hospital, Shanghai Institute of Ultrasound in Medicine, Shanghai, China; ^4^ Department of Ultrasound, Minhang Hospital, Fudan University, Shanghai, China; ^5^ Department of Ultrasonography, Fudan University Shanghai Cancer Center, Shanghai Medical College, Fudan University, Shanghai, China; ^6^ Department of Ultrasound, Shenzhen People’s Hospital, The Second Clinical Medical College, Jinan University, and The First Affiliated Hospital, Southern University of Science and Technology, Shenzhen, China; ^7^ Department of Ultrasound Medicine, The First Affiliated Hospital of Xi’an Jiaotong University, Xi’an, China; ^8^ Department of Ultrasound, People’s Hospital of Henan Province, Zhengzhou, China; ^9^ Department of Ultrasound Diseases, Tangdu Hospital, Four Military Medical University, Xi’an, China; ^10^ Department of Ultrasound, Sichuan Provincial People’s Hospital, University of Electronic Science and Technology of China, Chengdu, China; ^11^ Department of Ultrasound, Sun Yat-sen Memorial Hospital, Sun Yat-sen University, Guangzhou, China; ^12^ Department of Ultrasound, General Hospital of Northern Theater Command, Shenyang, China; ^13^ Department of Ultrasound, Affiliated Hangzhou First People’s Hospital, Zhejiang University School of Medicine, Hangzhou, China; ^14^ Department of Ultrasound, The Third Xiangya Hospital of Central South University, Changsha, China; ^15^ Department of Ultrasound, Peking University Third Hospital, Beijing, China; ^16^ Department of Ultrasound, Beijing Friendship Hospital, Capital Medical University, Beijing, China; ^17^ Department of Ultrasound, Affiliated Hospital of Guizhou Medical University, Guizhou, China; ^18^ Department of Ultrasound, Second Affiliated Hospital of Zhejiang University, School of Medicine, Hangzhou, China; ^19^ Department of Biostatistics, Institute of Medical Sciences, Shanghai Jiaotong University School of Medicine, Shanghai, China

**Keywords:** breast neoplasms, lymphatic metastasis, ultrasonography, elasticity imaging techniques, risk assessment

## Abstract

**Purpose:**

To develop a risk stratification system that can predict axillary lymph node (LN) metastasis in invasive breast cancer based on the combination of shear wave elastography (SWE) and conventional ultrasound.

**Materials and Methods:**

A total of 619 participants pathologically diagnosed with invasive breast cancer underwent breast ultrasound examinations were recruited from a multicenter of 17 hospitals in China from August 2016 to August 2017. Conventional ultrasound and SWE features were compared between positive and negative LN metastasis groups. The regression equation, the weighting, and the counting methods were used to predict axillary LN metastasis. The sensitivity, specificity, and the areas under the receiver operating characteristic curve (AUC) were calculated.

**Results:**

A significant difference was found in the Breast Imaging Reporting and Data System (BI-RADS) category, the “stiff rim” sign, minimum elastic modulus of the internal tumor and peritumor region of 3 mm between positive and negative LN groups (*p* < 0.05 for all). There was no significant difference in the diagnostic performance of the regression equation, the weighting, and the counting methods (p > 0.05 for all). Using the counting method, a 0–4 grade risk stratification system based on the four characteristics was established, which yielded an AUC of 0.656 (95% CI, 0.617–0.693, p < 0.001), a sensitivity of 54.60% (95% CI, 46.9%–62.1%), and a specificity of 68.99% (95% CI, 64.5%–73.3%) in predicting axillary LN metastasis.

**Conclusion:**

A 0–4 grade risk stratification system was developed based on SWE characteristics and BI-RADS categories, and this system has the potential to predict axillary LN metastases in invasive breast cancer.

## Introduction

Female breast cancer, the first cause of the death in malignant tumors (1), is the most common neoplasm in 20–59-year-old women; early diagnosis can reduce 40% of deaths (2–5). According to the American Joint Committee on Cancer, tumor size (T), node (N), and distant metastasis (M) have served as the global standard for conveying disease status among clinicians (6). Sentinel lymph node biopsy (SLNB) is regarded as the standard method for identifying the staging and determining the clinical arrangement. However, SLNB was recommended only for T1 or T2 tumors (7, 8), and multiple factors contribute to its limited pathological findings, suggesting that tedious and time-consuming SLNB is not optimal (9). Besides, SLNB may cause complications like arm numbness or upper limb edema in 3.5%–10.9% patients because of the increasing anesthesia time (10). Thus, an accurate and non-invasive examination for evaluating axillary LN status is expected before surgery or clinical treatment. However, showing a great difference among various studies, the sensitivity and specificity of axillary ultrasound in detection of LN metastasis ranged from 45.2% to 92.7% and from 40.5% to 93.9%, respectively (11–14).

For breast ultrasound examinations, as the suspicious imaging features increase, the Breast Imaging Reporting and Data System (BI-RADS) categories of the breast tumor increase accordingly. Tumors in BI-RADS category 4 or 5 indicate a risk of malignancy, and a detailed assessment of the SLN status is required (15). Shear wave elastography (SWE) is a novel ultrasonography based on the speed of shear wave velocity in different tissues, which can provide quantitative (that is kPa or m/s) and qualitative information (such as “stiff rim” sign) in differentiation of breast tumors (10, 12, 16). It has been reported that LN metastasis of breast cancer was associated with breast lesion elastic modulus (17). Besides, the “stiff rim” sign, usually appearing in malignant breast lesions with increased peritumoral stiffness, was regarded as the infiltration of cancer cells into the interstitial tissues or a desmoplastic reaction (16), which is an independent prognostic factor predicting tumor recurrence and patient death (18, 19). Thus, it is reasonable to hypothesize that the “stiff rim” sign may be potentially associated with LN metastasis. However, to our best knowledge, no study has detected the relationship between SWE information of peritumor tissues and axillary LN metastasis.

Hence, our study aims at assessing the risk of breast cancer LN metastasis in ultrasound examination by combining quantitative and qualitative SWE features, as well as BI-RADS categories.

## Materials and Methods

### Patients

This prospective study was approved by the institutional ethics committee, and written informed consent to participate was acquired before examinations. From August 2016 to August 2017, there were a total of 689 patients with malignant breast tumors from a multicenter consisting of 17 hospitals in China diagnosed by surgical pathology enrollment, with conventional ultrasound and SWE images acquired before treatment. The pathology of LNs from SLNB or axillary LN dissection was acquired. All macrometastases, micrometastases, and isolated tumor cells were counted as node positive. The exclusion criteria were set as follows: (1) patients with non-invasive malignant tumors according to the pathology; (2) patients who accepted chemotherapy before surgery; and (3) patients who accepted chemotherapy or biopsy before ultrasound examination. In total, we have excluded 70 patients, including 34 ductal carcinoma *in situ*, 11 solid papillary carcinoma, 6 intraductal papillary carcinoma, 2 cases with neoadjuvant chemotherapy before surgery, 6 cases of secondary tumor, and 11 cases without LN pathology. According to the rules established by the study, only one lesion was evaluated per patient, i.e., the most suspicious lesion in ultrasound examination or the largest one among the same BI-RADS category was selected in patients with multiple masses. Finally, our study included 619 invasive breast cancer participants (age range, 22–91 y; mean age, 52.16 + 11.21 y), including 1 male and 618 female patients.

### SWE Image Acquisition and Analysis

All the ultrasound examinations of patients from 17 hospitals used the Resona 7 ultrasound system (Mindray Medical International, Shenzhen, China) equipped with an L11-3 high-frequency probe. Prior to collecting data, all participating radiologists received systematic training in conventional ultrasound and SWE breast examination. Moreover, all radiologists from the multicenter had more than 3 years of experience in breast ultrasound elastography. Following standard conventional ultrasound examination, SWE was performed according to the already well-established method (20). The SWE display scale was set at 140 kPa according to the protocol provided by the manufacturer.

**Figure 1:**
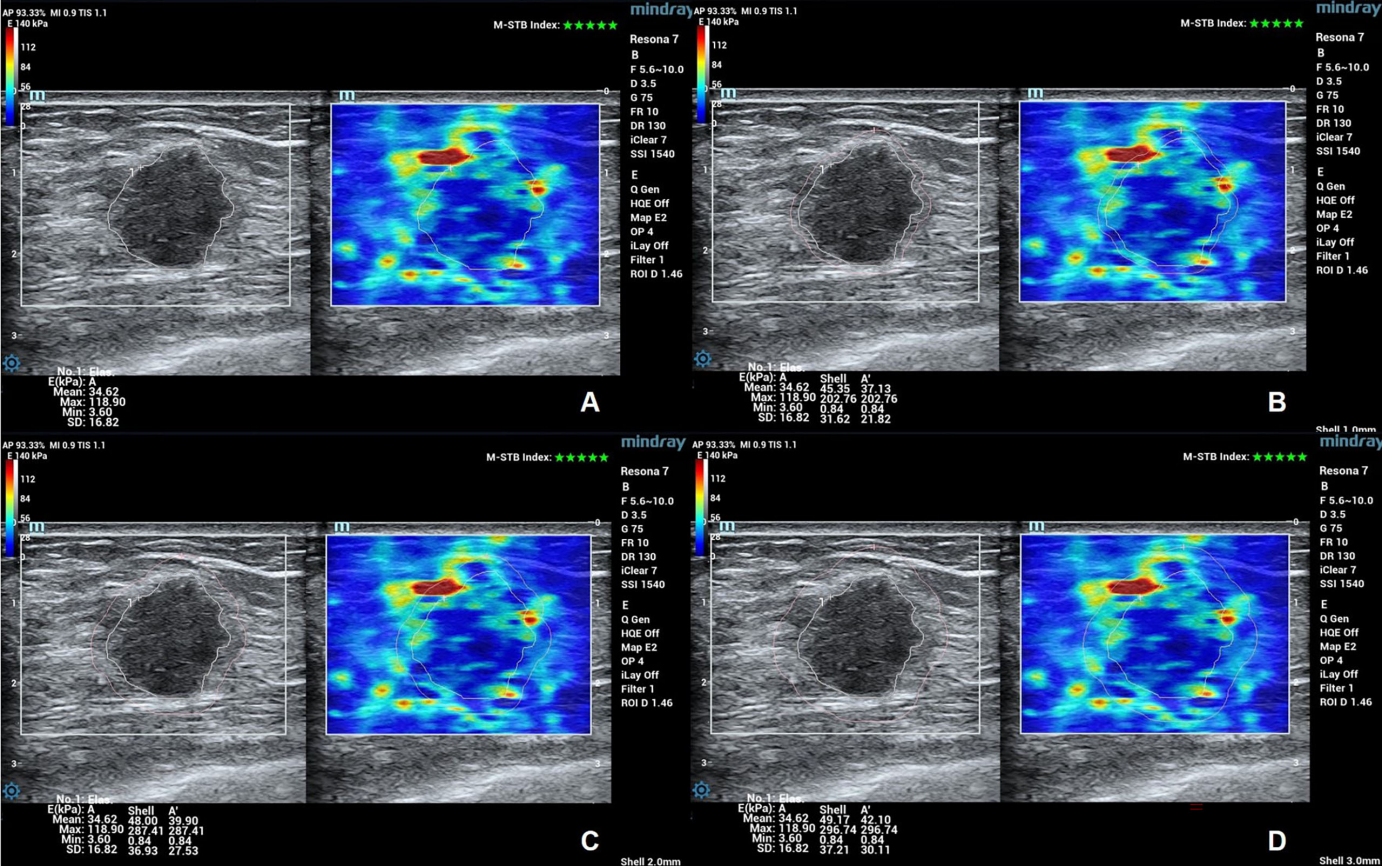
B-mode and SWE images of a patient with invasive breast tumor. The “stiff rim” sign presents on the SWE images. **(A)** SWE images and parameters of outlined tumor. The *E_max_*, *E_mean_*, *E_min_*, and *E_sd_* of internal tumor were 34.63, 118.90, 3.60, and 16.82 kPa. **(B)** SWE images and parameters of shell 1 mm. The *E_max_*, *E_mean_*, *E_min_*, and *E_sd_* of shell 1 mm were 45.35, 202.76, 0.84, and 31.62 kPa. **(C)** SWE images and parameters of shell 2 mm. The *E_max_*, *E_mean_*, *E_min_*, and *E_sd_* of shell 2 mm were 48.00, 287.41, 0.84, and 36.93 kPa. **(D)** SWE images and parameters of shell 3 mm. The *E_max_*, *E_mean_*, *E_min_*, and *E_sd_* of shell 3 mm were 49.17, 296.74, 0.84, and 37.21 kPa.

An online measurement of the tumor size and SWE parameters was done instantly by the examining radiologist. In measuring SWE parameters, the outline of the lesion was drawn by tracing, and the function of the “shell” was equipped to acquire the elastic modulus of the peritumoral tissues in regions of 1, 2, and 3 mm outside the boundary of internal tumors. Values of mean elasticity (*E_mean_*), maximum elasticity (*E_max_*), minimum elastic modulus (*E_min_*), standard deviation (*E_sd_*) evaluating both internal tumors and peritumoral tissues as well as intratumor ratio between mean elastic modulus of breast lesions, and normal fatty tissue (*E_ratio_*) were acquired and recorded. In assessing the location of the breast tumor, it is based on the location of the center of the lump in the 4 quadrants of the breast (upper/lower outer/inner quadrants). Upon completion of the evaluation, both B-mode images and SWE images of each patient were stored in a hard disk of the ultrasound system and subsequently sent to the study center (**Figure 1**).

At the study center, three radiologists with more than 8 years of experience in breast ultrasound evaluated ultrasound images according to consensus principles and proposed BI-RADS classification based on conventional ultrasound. At the same time, the presence of the “stiff rim” sign of the tumor was determined based on the SWE images. SWE images are colored in blue, green, orange, and red to sequentially show the progressive increase in tissue stiffness. When compared with the stiffness of the surrounding normal breast tissues and the interior tumor tissues, the peritumoral region, up to approximately 3 mm outside the boundary of internal tumors, showed increased stiffness (coded in orange or red), then it is judged as positive for the “stiff rim” sign (16).

### Statistical Analysis

Statistical analyses were performed using SPSS Version 23.0 (IBM, Armonk, NY, USA) and SAS 9.4(Statistical Analysis System, v.9.4). A p value less than 0.05 was considered for significant differences. The Mann–Whitney U test was used to compare the statistical difference in continuous variables between positive and negative LN groups, and the chi-square test was used to verify whether there was a statistical difference in categorical variables between the two groups. Significant variables were included in the final logistic regression analysis. The selection criteria alpha was set to 0.05. The odds ratio (OR) and 95% confidence interval (CI) for each feature were obtained from the logistic regression model. To predict axillary LN metastasis, the regression equation, the weighting, and the counting methods were used. The OR for the BI-RADS category and each SWE feature from the final logistic regression model was used in the weighting and counting methods. In the weighting method, the ORs were standardized using rounding to ensure appropriate application. In the counting method, the number of features with OR >1 was counted (21). The area under the receiver operating characteristic curve (AUC), sensitivity, specificity, and 95% CI of different variables were calculated, with histologic diagnosis as the gold standard. Finally, the risk system was established by balancing the diagnostic performance and ease of use. Multivariate logistic analysis was used to evaluate the OR of each risk grade. The Cochran–Armitage test was used to determine the change in the probability of axillary LN metastasis with the risk grade increased.

## Results

### Pathologic Results

A total of 619 invasive breast cancers included 508 cases of invasive ductal carcinoma and 111 cases of other types (**Table 1**). Among them, 174 (28.1%) patients were diagnosed with positive LN metastasis and 445 (71.9%) were negative. The mean size of the tumor was 20.99 ± 9.04 mm (range 4.7–89 mm).

**Table 1:** Pathologic diagnosis in 619 patients.

Histological type	Number
Invasive ductal carcinoma	508
Invasive lobular carcinoma	70
Mucinous breast carcinoma	9
Invasive ductal carcinoma with neuroendocrine differentiation	2
Metaplastic breast carcinoma	2
Invasive adenocarcinoma of breast	1
Invasive apocrine carcinoma	1
Tubular carcinoma	1
Others	
Intraductal carcinoma associated with microinvasive carcinoma	18
Invasive breast carcinoma with micropapillary features	5
Intracystic papillary breast carcinoma with areas of infiltration	1
Intraductal carcinoma associated with metaplastic squamous cell carcinoma	1

### Clinical and BI-RADS Characteristics

Features of categorical variables including staging of tumor, tumor location, and tumor BI-RADS categories were compared between negative and positive LN groups, as shown in **Table 2**. There was a significant difference in constituent ratio between 5 BI-RADS categories (*p* < 0.001). In the 619 masses, 481 lesions were defined as BI-RADS 3-4C, with about 25% (ranging from 22.4% to 27.3%) cases in positive metastasis LN, while in 138 masses of BI-RADS 5, patients with positive LN metastasis were up to 43.5%. There was no significant difference in tumor staging (*p* = 0.340) and location (*p* = 0.390) in the two groups.

**Table 2:** Clinical and ultrasound characteristics between negative and positive LN groups.

Parameters	Negative LN (n = 445)	Positive LN (n = 174)	Total (n = 619)	*p* value
Tumor staging				0.340
T1	244 (73.5%)	88 (26.5%)	332	
T2 and T3	201 (70.0%)	86 (30.0%)	287	
Tumor location				0.390
Upper outer quadrant	263 (72.3%)	101 (27.7%)	364	
Upper inner quadrant	106 (75.7%)	34 (24.3%)	140	
Lower outer quadrant	55 (65.5%)	29 (34.5%)	84	
Lower inner quadrant	21 (67.7%)	10 (32.3%)	31	
BI-RADS categories				*p* < 0.001
3	8 (72.7%)	3 (27.3%)	11	
4A	57 (75.0%)	19 (25.0%)	76	
4B	111 (75.0%)	37 (25.0%)	148	
4C	191 (77.6%)	55 (22.4%)	246	
5	78 (56.5%)	60 (43.5%)	138	
“Stiff rim” sign in SWE				*p* < 0.001
Present	304 (68.0%)	143 (32.0%)	447	
Absent	141 (82.0%)	31 (18.0%)	172	

BI-RADS, Breast Imaging Reporting and Data System; LN, lymph node; SWE, shear wave elastography.

### Qualitative SWE Characteristics

For the qualitative SWE feature, i.e., the “stiff rim” sign, there was a significant difference between the positive and negative groups for lymph node metastasis (*p* < 0.001). In patients with negative LN metastasis, 68.3% (304/445) cases presented a “stiff rim” sign in SWE images, while in positive LN groups, up to 82.2% (143/174) cases presented a “stiff rim” sign, significantly higher than that of negative LN groups.

### Quantitative SWE Characteristics

The characteristics of continuous SWE parameters in internal tumors and peritumor tissues between the two groups are shown in **Table 3**. The *E_mean_*, *E_max_*, *E_min_*, *E_sd_*, and *E_ratio_* of internal tumors in positive LN groups were similar to that of negative LN groups (*p* > 0.05 for all). The *E_mean_*, *E_max_*, and *E_sd_* of shell 1, 2, and 3 mm as well as *E_min_* of shell 1 and 2 mm showed no significant difference between the two groups (*p* > 0.05 for all). However, the *E_min_* of internal tumor (*p* = 0.005) and shell 3 mm (*p* = 0.007) was significantly lower in positive LN groups than that in the negative group. The AUC, sensitivity, specificity, 95% CI, and cutoff values are shown in **Table 4**. Only *E_min_* of internal tumor (*p <*0.01) and shell 3 mm (*p* < 0.01) could predict axillary LN metastasis, with cutoff values of 3.31 and 3.52, respectively.

**Table 3:** Quantitative evaluation of SWE in the internal and peritumor tissues between positive and negative LN metastasis groups.

	Negative LN (n = 445)	Positive LN (n = 174)	*p* value
SWE parameters of the tumor			
*E_mean_* (kPa)	24.50 (17.88–32.89)	23.66 (18.00–34.07)	0.754
*E_max_* (kPa)	115.03 (79.18–165.53)	110.05 (73.31–163.98)	0.743
*E_min_* (kPa)	3.64 (1.67–5.41)	2.67 (1.18–4.79)	0.005
*E_sd_* (kPa)	15.02 (10.67–21.64)	14.67 (9.35–22.11)	0.344
*E_ratio_*	5.61 (4.20–7.31)	5.24 (4.27–7.39)	0.579
Shell of tumor			
1 mm			
*E_mean_* (kPa)	34.47 (25.88–47.87)	33.34 (25.75–47.33)	0.564
*E_max_* (kPa)	140.54 (97.98–207.07)	131.76 (89.86–194.43)	0.237
*E_min_* (kPa)	3.23 (1.05–5.65)	2.70 (1.01–5.35)	0.281
*E_sd_* (kPa)	22.92 (15.87–31.86)	21.91 (14.78–30.08)	0.336
2 mm			
*E_mean_* (kPa)	36.02 (26.27–47.75)	34.73 (25.13–47.18)	0.523
*E_max_* (kPa)	147.05 (105.21–212.23)	146.00 (100.27–199.41)	0.500
*E_min_* (kPa)	2.72 (0.90–5.27)	2.32 (0.83–4.39)	0.141
*E_sd_* (kPa)	22.63 (16.79–32.33)	22.11 (15.13–30.31)	0.351
3 mm			
*E_mean_* (kPa)	35.97 (25.60–45.77)	34.64 (23.77–45.69)	0.495
*E_max_* (kPa)	147.68 (104.28–209.37)	146.00 (101.97–202.73)	0.775
*E_min_* (kPa)	2.65 (0.98–5.40)	2.03 (0.66–3.55)	0.007
*E_sd_* (kPa)	22.23 (16.27–31.31)	21.42 (15.43–28.84)	0.238

All data represent as median with interquartile range in parentheses. E_mean_, mean elastic modulus; E_max_, the maximum elastic modulus; E_min_, minimum elastic modulus; E_sd_, elastic modulus standard deviation; E_ratio_, ratio between mean elastic modulus of breast lesions and normal fatty tissue; LN, lymph node; SWE, shear wave elastography.

**Table 4:** Quantitative SWE evaluation of the tumor and peritumor shell for the predicting of axillary LN metastasis.

	Sensitivity (%) (95% CI)	Specificity (%) (95% CI)		Cutoff value	AUC (95% CI)	*p* value
SWE parameters of the tumor						
*E_mean_* (kPa)	54.02 (46.3–61.6)	51.69 (46.9–56.4)		≤24.16	0.508 (0.468–0.548)	0.756
*E_max_* (kPa)	4.60 (2.0–8.9)	89.89 (86.7–92.5)		≤49.43	0.508 (0.468–0.549)	0.745
*E_min_* (kPa)	59.77 (52.1–67.1)	55.96 (51.2–60.6)		≤3.31	0.573 (0.533–0.613)	< 0.01
*E_sd_* (kPa)	27.59 (21.1–34.9)	80.67 (76.7–84.2)		≤9.91	0.524 (0.484–0.564)	0.352
*E_ratio_*	50.57 (42.9–58.2)	56.40 (51.7–61.1)		≤5.24	0.514 (0.474–0.554)	0.582
Shell of tumor						
1 mm						
*E_mean_* (kPa)	44.83 (37.3–52.5)	62.02 (57.3–66.6)		≤30.90	0.515 (0.475–0.555)	0.567
*E_max_* (kPa)	27.01 (46.3–61.6)	79.78 (75.7–83.4)		≤90.87	0.531 (0.490–0.570)	0.234
*E_min_* (kPa)	63.22 (55.6–70.4)	45.84 (41.1–50.6)		≤3.67	0.528 (0.488–0.568)	0.282
*E_sd_* (kPa)	40.23 (32.9–47.9)	66.29 (61.7–70.7)		≤18.34	0.525 (0.485–0.565)	0.338
2 mm						
*E_mean_* (kPa)	27.59 (21.1–34.9)	77.98 (73.8–81.7)		≤25.51	0.517 (0.476–0.557)	0.525
*E_max_* (kPa)	74.14 (67.0–80.5)	32.13 (27.8–36.7)		≤193.19	0.517 (0.477–0.557)	0.498
*E_min_* (kPa)	67.82 (60.3–74.7)	42.92 (38.3–47.7)		≤3.38	0.538 (0.498–0.578)	0.134
*E_sd_* (kPa)	47.13 (39.5–54.8)	60.22 (55.5–64.8)		≤20.59	0.524 (0.484–0.564)	0.353
3 mm						
*E_mean_* (kPa)	27.59 (21.1–34.9)	78.43 (74.3–82.2)		≤24.61	0.518 (0.477–0.558)	0.497
*E_max_* (kPa)	82.76 (76.3–88.1)	22.47 (18.7–26.6)		≤218.08	0.507 (0.467–0.547)	0.772
*E_min_* (kPa)	75.29 (68.2–81.5)	39.78 (35.2–44.5)		≤3.52	0.570 (0.530–0.609)	< 0.01
*E_sd_* (kPa)	37.93 (30.7–45.6)	70.11 (65.6–74.3)		≤17.85	0.530 (0.490–0.570)	0.238

AUC, area under curve; CI, confidence interval; E_mean_, mean elastic modulus; E_max_, the maximum elastic modulus; E_min_, minimum elastic modulus; E_sd_, elastic modulus standard deviation; E_ratio_, ratio between mean elastic modulus of breast lesions and normal fatty tissue; LN, lymph node; SWE, shear wave elastography.

### Risk Grade of LN Metastasis and Its Diagnostic Performance

**Figure 2:**
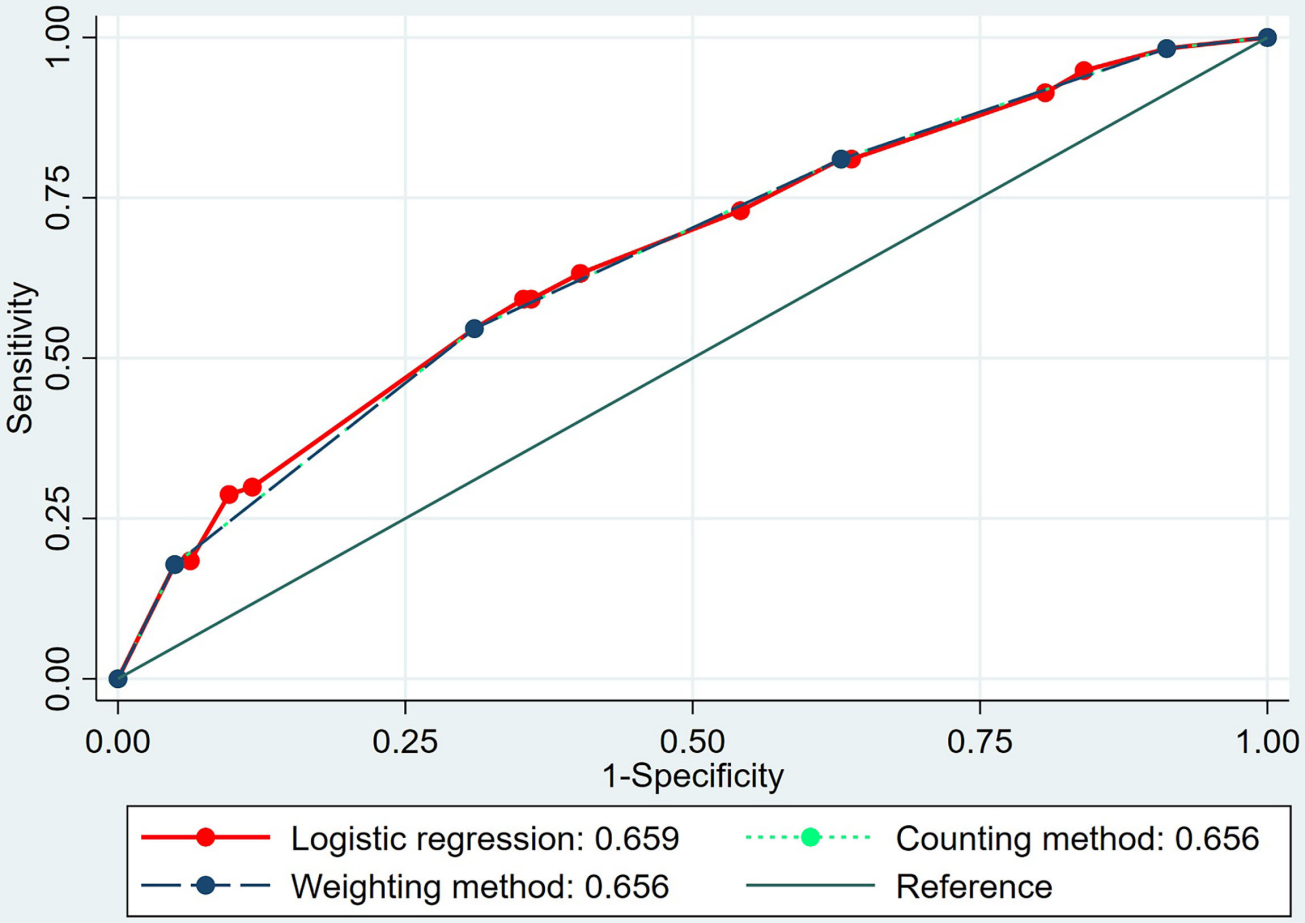
The area under the receiver operating characteristic (ROC) curves of the logistic regression equation, the weighting method, and the counting method in predicting axillary lymph node metastasis of invasive breast cancer.

The multiple logistic regression analysis indicated that the following four features were significantly associated with LN metastasis: (1) the breast tumor was assessed as BI-RADS 5; (2) the *E_min_* of intrinsic tumor was no more than 3.31 kPa; (3) the *E_min_* of shell 3 mm was no more than 3.52 kPa; and (4) the tumor showed a positive “stiff rim” sign in SWE. The OR values of the four features were 2.155, 1.654, 1.564, and 1.900, respectively (*p* < 0.05 for all). In the weighting method, the weight values of all four features were 2 based on the OR values of the above four features (**Table 5**). In the prediction of LN metastasis, the logistic regression equation yielded the highest AUC of 0.659 (95% CI, 0.621–0.697), while the weighting method and the counting method had the same AUC of 0.656 (95% CI, 0.617–0.693). There was no statistical difference among the three (p > 0.05 for all) (**Table 6** and **Figure 2**).

**Table 5:** Odds ratios for the suspicious features between positive and negative LN groups and the corresponding weighting values.

Suspicious features	Logistic regression		
	OR (95% CI)	*p* value*^a^*	Weighting value
BI-RADS categories			
3–4C	1.00 (reference)		1
5	2.155 (1.431–3.247)	<0.05	2
*E_min_*			
>3.31	1.00 (reference)	<0.05	1
≤3.31	1.654 (1.120–2.442)		2
*E_min_* of shell 3 mm			
>3.52	1.00 (reference)		1
≤3.52	1.564 (1.019–2.401)	<0.05	2
“Stiff rim” sign in SWE			
Present	1.00 (reference)		1
Absent	1.900 (1.210–2.982)	<0.05	2

BI-RADS, Breast Imaging Reporting and Data System; E_min_, minimum elastic modulus; LN, lymph node; OR, odds ratios; SWE, shear wave elastography.

^a^Compared with reference.

**Table 6:** Comparison of the logistic regression equation, the weighting, and the counting methods in predicting the axillary LN metastasis.

Method	Sensitivity (%) (95% CI)	Specificity (%) (95% CI)	AUC (95% CI)
Logistic regression equation	59.20 (51.5–66.6)	64.72 (60.1–69.2)	0.659 (0.621–0.697)
The weighting method	54.60 (46.9–62.1)	68.99 (64.5–73.3)	0.656 (0.617–0.693)
The counting method	54.60 (46.9–62.1)	68.99 (64.5–73.3)	0.656 (0.617–0.693)

AUC, area under curve; CI, confidence interval; LN, lymph node.

Following the principle of balancing the diagnostic performance and ease of use, the counting method was used to establish the risk system for predicting axillary LN metastases. Thus, a 0–4 grade risk stratification system was established based on the four suspicious features mentioned above: The LN metastasis risk grade of the breast cancer is defined from 0 to 4 according to the number of suspicious features, i.e., the mass was judged to be risk grade 4 when all suspicious features were present and grade 0 when no suspicious features were present. The risk grade of LN metastasis for the 619 patients was determined according to the above rule.

**Figure 3:**
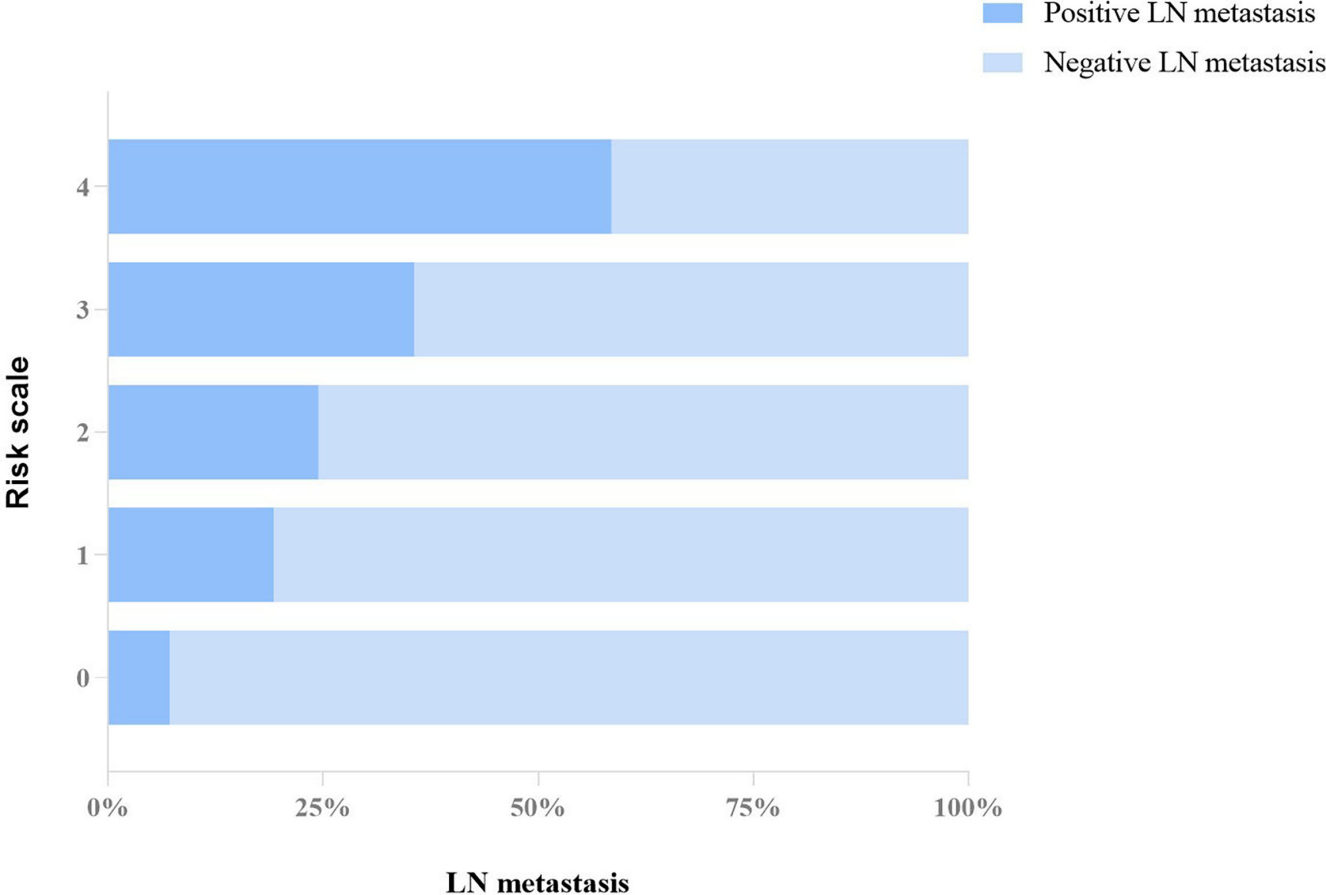
Proportion distribution of positive and negative LN metastasis in each grade of the risk system.

Subsequently, we compared the difference of risk grade between patients with positive and negative metastasis LNs (**Table 7**), and a significant difference was shown between the two groups (*p* < 0.001). The percentage of patients with positive LNs from grades 0 to 4 was constantly increased, with 7.1% (3/42) for grade 0, 19.2% (30/156) for grade 1, 24.5% (46/188) for grade 2, 35.6% (64/180) for grade 3, and 58.5% (31/53) for grade 4, respectively, which is shown in **Figure 3**. The Cochran–Armitage trend test indicated that the risk of axillary LN metastasis was increased with the increase in grade level (*p* < 0.0001). Results of the logistic analysis of the comprehensive grade showed that the OR of axillary LN metastasis was increased progressively with rising grade. The risk of axillary LN metastasis was 3.095 times higher in patients with risk grade 1 than in patients with risk grade 0 (p = 0.074), and 4.211–18.318 times higher in patients with risk grades 2–4 (p < 0.05 for all). The risk grade model predicted axillary LN metastasis with an AUC of 0.656 (95% CI, 0.617–0.693), a sensitivity of 54.60% (95% CI, 46.9%–62.1%), a specificity of 68.99% (95% CI, 64.5%–73.3%), and a cutoff value of grade 2.

**Table 7:** Proportion and odds ratios for each risk grade in negative and positive LN metastasis groups.

Risk grade	Negative LN (n = 445)	Positive LN (n = 174)	OR	*p* value*^a^*
0	39 (92.9%)	3 (7.1%)	1.00 (reference)	–
1	126 (80.8%)	30 (19.2%)	3.095 (0.896–10.696)	0.074
2	142 (75.5%)	46 (24.5%)	4.211 (1.243–14.271)	0.021
3	116 (64.4%)	64 (35.6%)	7.172 (2.132–24.132)	0.001
4	22 (41.5%)	31 (58.5%)	18.318 (5.016–66.892)	<0.001

LN, lymph node; OR, odds ratios.

^a^Compared with reference.

Since risk category 1 was not different from category 0, we also compared the difference of OR values between grade 1 vs. grades 2, 3, and 4 and grades 0–1 vs. grades 2, 3, and 4 in the risk system. Results showed that the risk of axillary LN metastasis was higher in patients with risk grades 3 and 4 than in patients with risk grades 0–1 and grade 1 (p < 0.05 for all) (**Table 8**).

**Table 8:** Differences of odds ratios between grade 1 and grades 2, 3, 4, as well as between grades 0–1 and grades 2, 3, 4.

Grade group	OR	*p* value*^a^*
Grade 1 vs. grade 2, 3, 4		
1	1.00 (reference)	–
2	1.361 (0.810–2.286)	0.245
3	2.317 (1.403–3.827)	< 0.01
4	5.918 (3.010–11.636)	< 0.01
Grade 0–1 vs. grade 2, 3, 4		
0–1	1.00 (reference)	–
2	1.620 (0.982–2.671)	0.059
3	2.759 (1.703–4.469)	< 0.01
4	7.045 (3.634–13.659)	< 0.01

LN, lymph node; OR, odds ratios.

^a^Compared with reference.

## Discussion

The risk of LN metastasis constantly exists in breast cancer patients, and it is expected to be provided in an early stage in order to guide the clinical decision and improve the tumor survival. SWE has been used to predict axillary LN metastasis in invasive breast cancer. Previous studies have reported that higher elasticity of the tumor was related to lymph node metastasis of breast cancer (22, 23), and further study has revealed that the mean elastic modulus of internal breast tumor could independently predict axillary LN metastasis (17). Wen et al. reported that the *E_max_* and *E_mean_* of primary tumors were higher in LN metastasis cases (24). In our study, however, quantitative SWE parameters including *E_mean_*, *E_max_*, *E_sd_*, and *E_ratio_* did not show a significant difference between patients with positive and negative LN metastasis groups (*p* > 0.05 for all), indicating that these SWE parameters of intrinsic tumor and peritumor tissues might not provide sufficient information in predicting LN metastasis for our research group. The difference between the results of this study and previous studies may be due to the difference of the study sample and the difference of the ultrasound instruments used.

In our study, SWE parameters including the *E_min_* of internal tumor and shell 3 mm and the “stiff rim” sign showed a significant difference between patients with positive and negative LN metastasis groups (*p* < 0.05 for all). Zhou et al. have reported that the “stiff rim” sign could select the malignant breast lesions, and possible reasons may contribute to cancer cell infiltration into interstitial tissues or a desmoplastic reaction (16), indicating that a qualitative assessment of peritumoral tissue stiffness may reflect the progress of invasive malignant tumors to some extent. Peritumoral stroma and peritumoral invasion played a critical role in the spreading and metastasis of breast cancer (18, 19, 25, 26) and were also responsible for the formation of the “stiff rim” sign, as well as the attenuation of shear wave energy (16). The low-quality shear waves caused by attenuation may be interpreted as slow-speed shear waves (16). Therefore, the “stiff rim” sign was mostly found in the positive LN metastasis group, and the *E_min_* of the positive LN metastasis group was lower than that of the negative LN metastatic group.

There was a significant difference in the constituent ratio between 5 BI-RADS categories (p < 0.001). In the 619 masses, 481 lesions were defined as BI-RADS 3-4C, with about 25% (ranging from 22.4% to 27.3%) cases in positive metastasis LN, while in 138 masses of BI-RADS 5, patients with positive LN metastasis were up to 43.5%. Guo et al. revealed that irregular shape and higher color Doppler flow imaging grades were associated with axillary LN metastasis of breast cancer (27). Bae et al. showed that architectural distortion of breast tumors was associated with LN metastasis of breast cancer (28). Previous studies also indicated that a non-circumscribed margin and heterogeneous internal echo were potential predictors of axillary LN metastasis independently (28, 29). Possible reasons might be that the non-circumscribed margins reflect the invasiveness of tumors and aggressive of metastatic lesions to lymphocytes. Besides, tumor growth rate might influence the internal echo of the masses, and rapid tumor growth could lead to an increased likelihood of LN metastasis (29). These ultrasound features, such as irregular shape, non-circumscribed margin, and architectural distortion, were considered as determining characteristics of ultrasound BI-RADS, which may explain the higher incidence of LN metastasis in BI-RADS 5 tumors in this study.

A previous study revealed that tumor staging was an independent predictor of breast cancer LN metastasis (30). A tumor size of 20 mm or greater on ultrasound was an independent predictor of LN metastasis (31). In the present study, however, there was no significant difference in the number of tumors evaluated as T1 and T2/T3 in LN-positive and -negative groups (*p* = 0.340), which was similar to the results of the previous study; that is, tumor size on ultrasound was not an independent predictor of axillary LN metastasis (28). Breast tumor location has been acknowledged as an independent factor of tumor prognosis, and malignant tumors in the lower inner quadrant were related to a worse prognosis because of the internal mammary lymphatic pathway without sufficient studies (32). However, there was no significant difference in tumor location (*p* = 0.390) in LN-positive and -negative groups in our study.

The grade system has been extensively used in categorical variables of clinical and imaging data analysis (1, 33). In the prediction of LN metastasis, there was no statistical difference among the logistic regression equation, the weighting method, and the counting method (p > 0.05 for all). For ease of use, a 0–4 grade risk stratification system for LN metastasis was established based on the counting method in this study. The risk system was based on quantitative and qualitative SWE features as well as BI-RADS category. To the best of our knowledge, no studies have combined quantitative and qualitative SWE characteristics of tumors, especially peritumor tissues, and the BI-RADS category to predict axillary LN metastasis in breast invasive cancer. Our results showed that the risk of axillary LN metastasis of breast cancer increased as the risk grade rises from 0 to 4, with the increase of the four significant factors (BI-RADS, “stiff rim” sign, *E_min_* of internal tumor, and shell 3 mm). A patient with a BI-RADS 5 breast tumor exhibiting the “stiff rim” sign (value of *E_min_* in internal tumor and shell 3 mm ≤3.31 and ≤3.52, respectively) was assessed as grade 4 in our risk system. The OR of each grade in the risk system gradually increased as the increasing levels were 3.095 (*p* = 0.074, 95% CI = 0.896–10.696) for grade 1, 4.211 (*p* < 0.05, 95% CI = 1.243–14.271) for grade 2, 7.172 (*p* < 0.05, 95% CI = 2.132–24.132) for grade 3, and 18.318 (*p* < 0.05, 95% CI = 5.016–66.892) for grade 4, respectively, compared with grade 0, indicating that patients with a higher risk grade were more likely to have LN metastasis. The diagnostic performance of this risk grade system is significant in assessing the risk grade of LN metastasis, although the AUC is not excellent (0.656, 95% CI, 0.617–0.693).

The merit of our research is that this is a multicenter study covering 17 hospitals, which effectively improves the reliability of the research data. However, the study still has some limitations. Firstly, the evaluation of the BI-RADS category and the SWE features of breast lesions were influenced by the experience of the radiologist. However, previous studies have confirmed that the reproducibility ranged from moderate to substantial for the BI-RADS category and ranged from substantial to almost perfect for both the quantitative SWE features and the “stiff rim” sign (16, 34, 35). Secondly, all SWE information were acquired from the same ultrasound system (Resona 7, Mindray), which would limit the generalizability of the study results. Further studies using different ultrasound SWE systems are warranted in the future. Third, the AUC of the risk system is not very high. In the future, better predictive models may be obtained if the information provided by ultrasound, MRI, mammography, and clinical data is utilized jointly.

## Conclusion

A 0–4 grade risk stratification system, based on quantitative and qualitative SWE features as well as BI-RADS category, for predicting axillary LN metastasis in invasive breast cancer was established in this study. Although the diagnostic performance of this risk system was not excellent, there is no doubt that it has the potential to predict axillary LN metastasis by the combination of SWE and conventional ultrasound.

## Data Availability Statement

The datasets presented in this article are not readily available because the generated datasets belong to department of ultrasound, Ruijin Hospital, Shanghai Jiaotong University School of Medicine. Requests to access the datasets should be directed to JQZ, zhousu30@126.com.

## Ethics Statement

The studies involving human participants were reviewed and approved by the Ethics Committee of Ruijin Hospital, Shanghai Jiaotong University School of Medicine. The patients/participants provided their written informed consent to participate in this study.

## Author Contributions

JWZ drafted and edited the manuscript. HTZ, YJD, XHJ, JWZ, ZYL, ZRC, YJX, BH, YXH, CC, JFX, FJD, XNX, CRW, WJH, GW, QYL, QC, WYD, QCJ, YLM, HNY, XJX, HJY, PZ, YS, LGC, PH, LXQ, JPL, LYS, YNZ, and YYX collected the clinical and ultrasound data. YJD and WWZ analyzed the images. XHJ collected the ultrasound images. XHJ and JWZ collected the autopsy and pathology data. HTZ prepared for the literature. YYS and HTZ completed the statistical analysis. All authors contributed to the article and approved the submitted version.

## Funding

This study was supported by the National Natural Science Foundation of China (grant no. 82071928).

## Conflict of Interest

The authors declare that the research was conducted in the absence of any commercial or financial relationships that could be construed as a potential conflict of interest.

## Publisher’s Note

All claims expressed in this article are solely those of the authors and do not necessarily represent those of their affiliated organizations, or those of the publisher, the editors and the reviewers. Any product that may be evaluated in this article, or claim that may be made by its manufacturer, is not guaranteed or endorsed by the publisher.
